# Sports and Exercise at Different Ages and Leukocyte Telomere Length in Later Life – Data from the Berlin Aging Study II (BASE-II)

**DOI:** 10.1371/journal.pone.0142131

**Published:** 2015-12-02

**Authors:** Denise Saßenroth, Antje Meyer, Bastian Salewsky, Martin Kroh, Kristina Norman, Elisabeth Steinhagen-Thiessen, Ilja Demuth

**Affiliations:** 1 Research Infrastructure 'Socio-Economic Panel (SOEP)' at the German Institute for Economic Research (DIW Berlin), Berlin, Germany; 2 Humboldt University (HUB), Berlin, Germany; 3 The Berlin Aging Study II; Research Group on Geriatrics, Charité –Universitätsmedizin Berlin, Berlin, Germany; 4 Institute of Medical and Human Genetics, Charité –Universitätsmedizin Berlin, Berlin, Germany; University of Newcastle, UNITED KINGDOM

## Abstract

Physical activity and sports have repeatedly been reported to be associated with telomere length. We studied the association of different types of sports across different stages of life on relative leukocyte telomere length (rLTL) in advanced age.815 participants (397 men) from the Berlin Aging Study II aged over 61 years were included in the analysis. rLTL was measured by real time PCR and physical activity was determined retrospectively by questionnaire, assessing type and duration of sports in the past as well as currently. Five separate multiple linear regression models adjusted for various control variables were performed. 67.3% of participants exercised currently, whereas 19.4% performed sports only between the age of 20 and 30. rLTL was higher in subjects who stated to exercise currently (N = 456), and in subjects who engaged in endurance (N = 138) or intensive activity sports (N = 32). Current physical activity was positively associated with rLTL in the risk factor adjusted regression model (β = 0.26, p < 0.001) and practicing sports for a minimum of 10 years preceding the assessment had a significant effect on rLTL (β = 0.39, p = 0.011). The highest impact was seen for intensive activity sports (β = 0.79, p < 0.001) and physical activity since at least 42 years (β = 0.47, p = 0.001). However, physical activity only between 20 and 30 years of age did not affect rLTL in old age when compared to no sports at all (β = -0.16, p = 0.21). Physical activity is clearly associated with longer rLTL. The effect is seen with longer periods of physical activity (at least 10 years), with intensive sports activities having the greatest impact on rLTL. Our data suggest that regular physical activity for at least 10 years is necessary to achieve a sustained effect on rLTL.

## Introduction

The ends of the linear chromosomes, telomeres, represent a highly organized structure consisting of a hexanucleotide repeat (in humans [TTAGGG]), the so called ‘core telomeric proteins’ of the shelterin complex, and a growing number of associated proteins with telomere related functions (reviewed in [1, 2]). It is widely accepted that telomere length, which can be estimated with different methods such as southern blot or quantitative PCR, reflects biological age when analyzed on the population level (reviewed in [3]).

Several cross-sectional studies report an inverse association between telomere length and various health related risk factors (e.g. [4–7]) and quite a few age related human diseases have been shown to be associated with shorter telomeres ([8–10]; reviewed in [11]). Mainly based on observations from cross-sectional studies, it is suggested that lifestyle associated risk factors impact telomere length in later life; however, little data supporting this assumption are available. Indeed, data from a 2014 study refute this assumption since no correlation of body weight, smoking status, physical activity, and alcohol intake with changes in telomere length during 10 years of observation was found, although cross-sectionally these lifestyle factors were significantly correlated with telomere length [7].

Among the repeatedly reported lifestyle related risk factors associated with shorter telomere length are increased body mass index (meta-analysis in [6]), increased waist-to-hip ratio (e.g. [4, 5, 12]), smoking (e.g. [5, 8]), alcohol consumption (e.g. [7, 13]) and physical inactivity [7, 14]. However, other studies have not found these associations (e.g. [7, 12, 15–17]), which might be due to differences in the studied cohorts and in the analysis methods used. While the association between physical activity/sport and telomere length is studied extensively using cross-sectional data, so far the effects of sport and exercise across different stages of life, related to telomere length in old age, is not examined. The current study addresses this question by analyzing a large sample of community-dwelling participants aged 61 years or older, drawn from the Berlin Aging Study II (BASE-II).

## Results

A total of 814 older BASE-II participants (age ranging between 61 and 82; a total of 397 men) were included in this analysis. Approximately 67.3% of the BASE-II respondents stated that they exercised currently, while 19.4% stated they only exercised regularly between 20 and 30 years of age (see Table 1).

**Table 1:** Descriptive Statistics of the Study Population.

Variables	Mean, standard deviation, respectively %	Total number of observations*
Relative telomere length (rLTL)	1.1 ± 0.2	814
Age (years)	68.8 ± 3.4	814
Male (%)	48.8	814
Married (%)	54.3	814
Father’s age at birth (years)	33.3 ± 6.6	732
Years of education	13.7 ± 3.8	814
Equivalized household income (EUR)	1841.321 ± 970.8	761
Body mass index	26.7 ± 4.3	814
Heavy alcohol intake (%)	27.5	811
Smoker (%)	7.2	809
Physically active (%)	67.3	810
Physically active only in the age of 20–30 (%)	19.4	444
Years since physical activity is practised	18.9 ± 22.2	787

*Total number of observation varies by variables due to item nonresponse.

**Fig 1:**
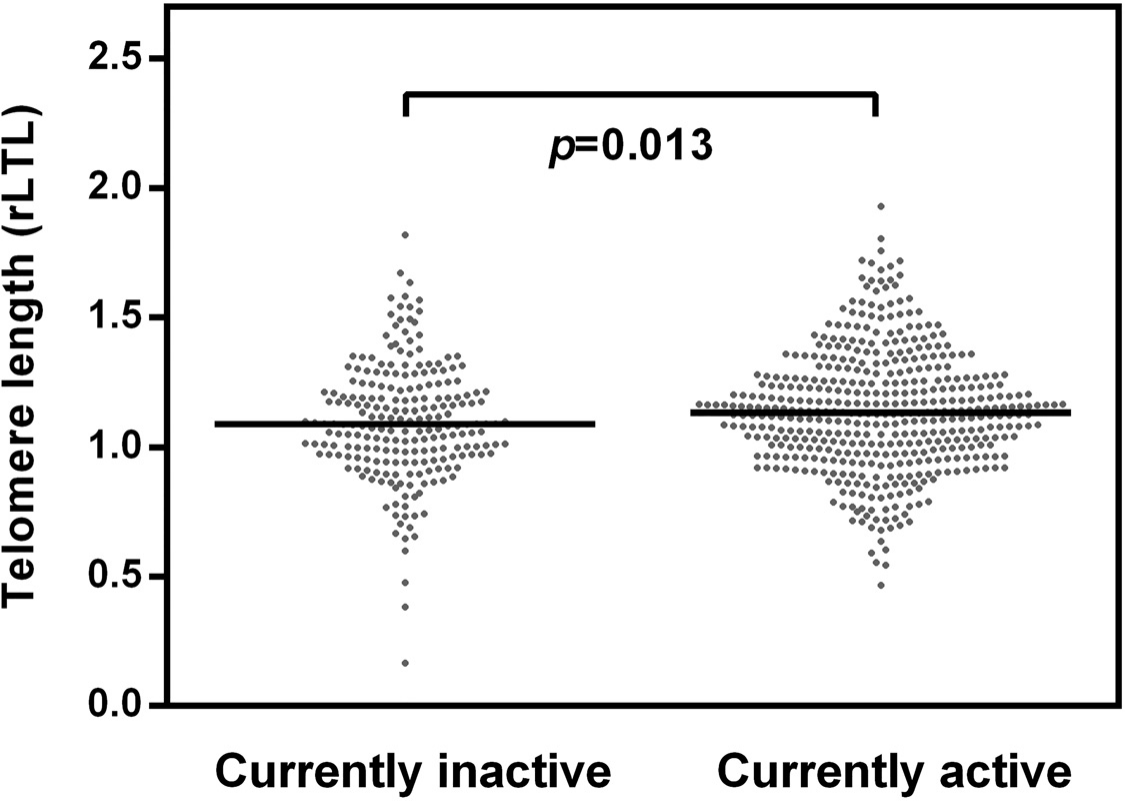
Standardized values of rLTL by current activity. Individuals of the currently physically active group showed significantly longer telomeres when compared to the inactive group (inactive group, N = 223; active group, N = 456). Two-tailed *p*-values were determined using the independent T-test.

**Fig 2:**
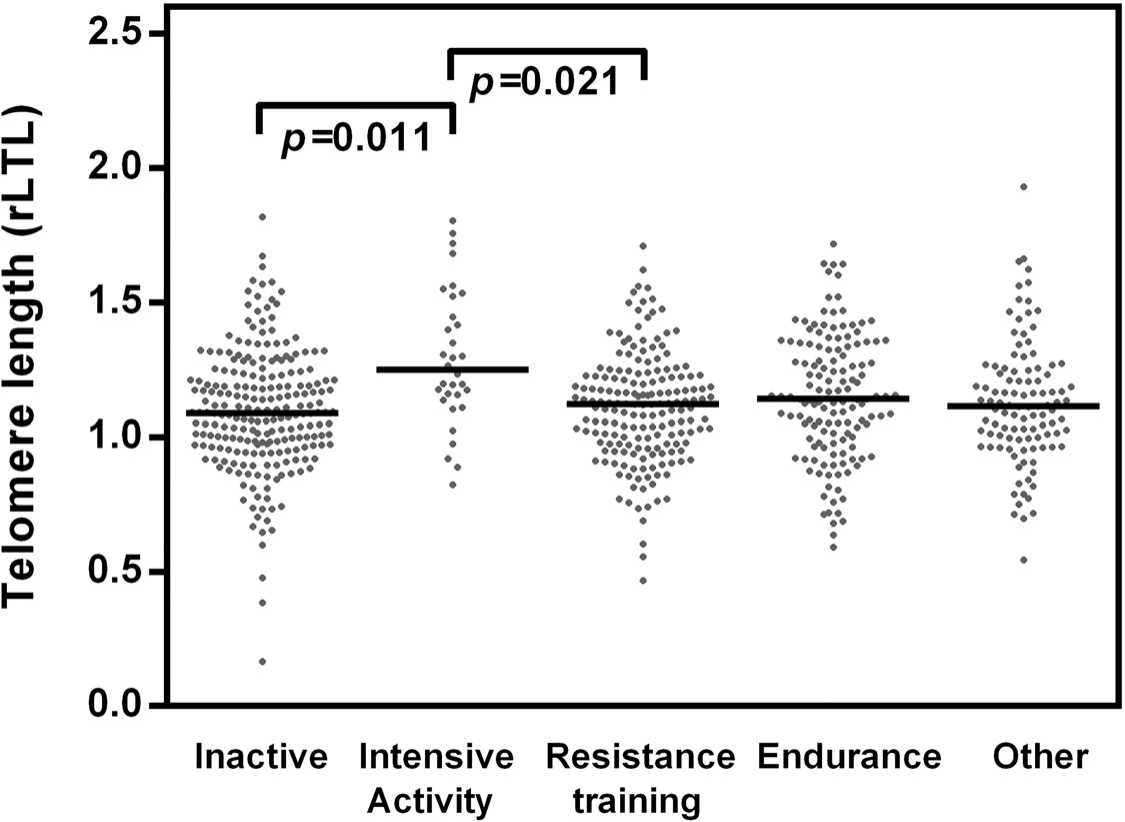
Standardized values of rLTL by type of sports currently practiced. Individuals of the different groups of sport types showed significant differences in telomere length (inactive group, N = 223; group intensive activity, N = 32; group resistance training, N = 177; group endurance, N = 138; group other type of sports, N = 106). The *p*-value was determined conducting one-way ANOVA. The *post hoc* Tukey’s test revealed significant differences for respondents engaged in intensive activity compared to inactive respondents (p = 0.011), and respondents who engaged in resistance training (p = 0.021).

**Fig 3:**
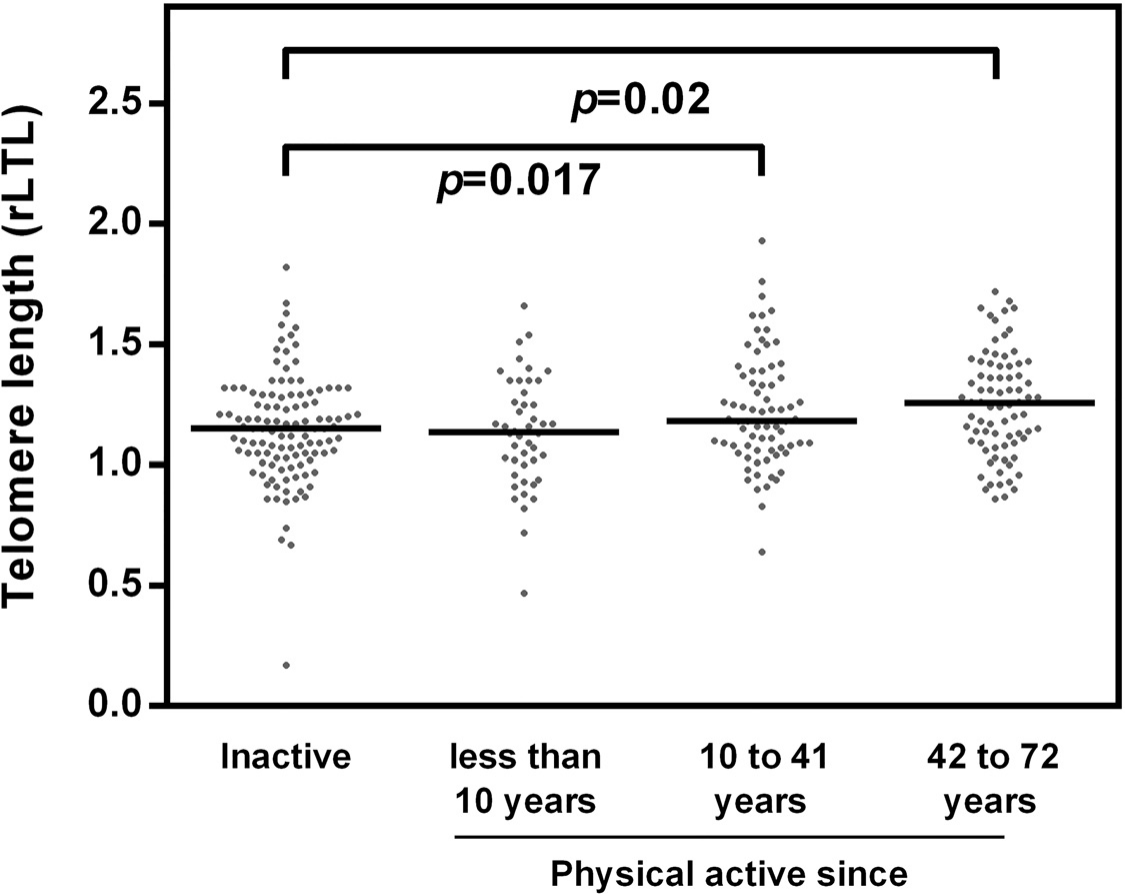
Standardized values of rLTL by exercise duration. Individuals of the different groups regarding duration since physical activity was practiced showed significant differences (p = 0.0048) in the length of telomeres (inactive group, N = 113; group active 1 to 9 years, N = 46; group active 10 to 41 years, N = 73; group active more than 41 years, N = 78). The *p*-value was determined conducting one-way ANOVA. The *post hoc* Tukey’s test revealed a significant difference between physical activity since more than 41 years and current inactivity (p = 0.017). Moreover relative telomere length of the group active more than 41 years differs significantly from the group physically active 1 to 9 years (p = 0.035).

**Fig 4:**
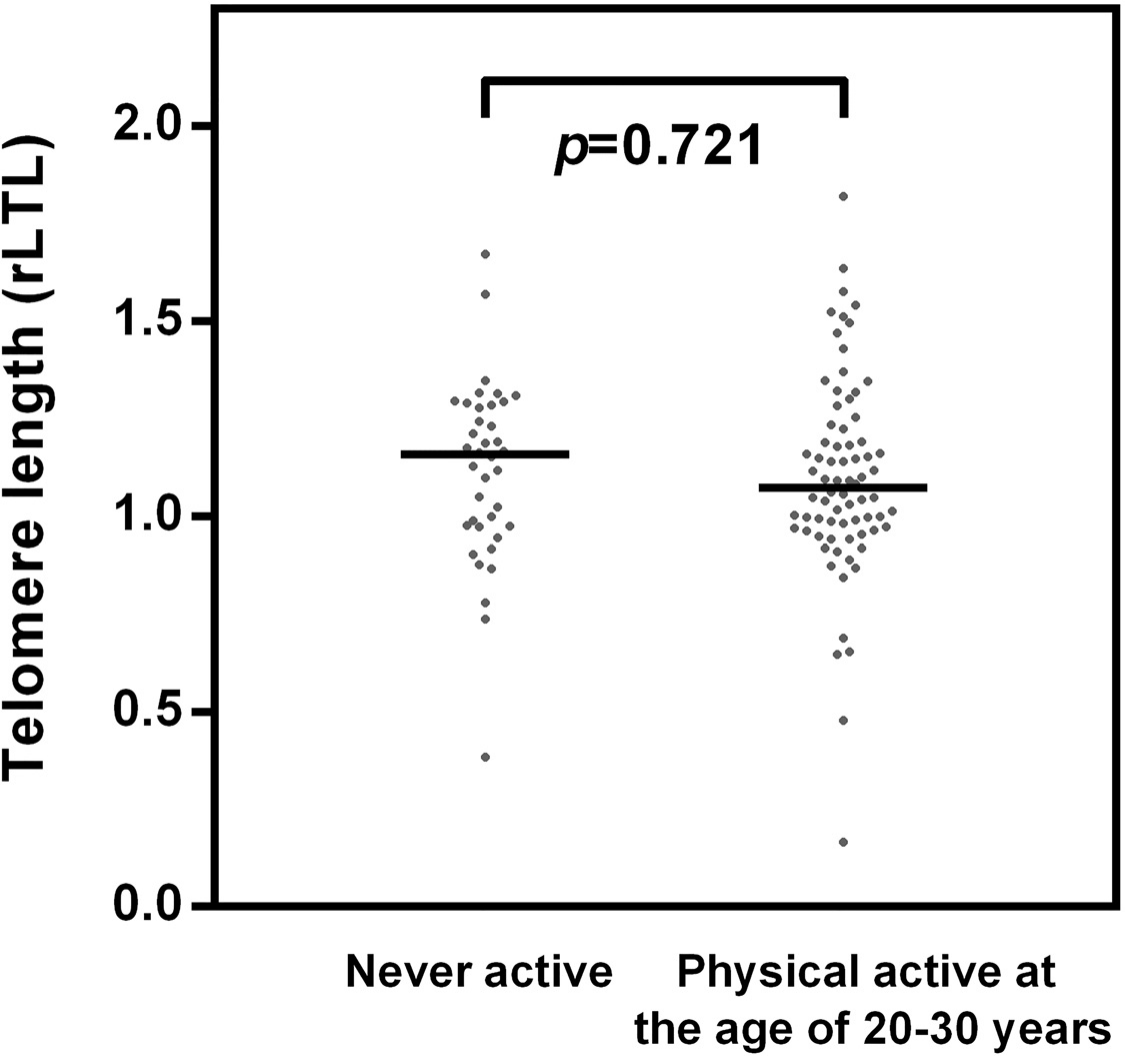
Standardized values of rLTL by sport in young age only. Individuals of the physically active group in the age 20 to 30 showed no significant differences in telomere length when compared to the inactive group which never engaged in sports (inactive group, N = 38; group physically active in young age only, N = 74). Two-tailed *p*-values were determined using the independent T-test.

Figs 1 to 4 show relative leukocyte telomere length according to different sport variables. Fig 1 displays the differences between rLTL for study participants who are currently active and those who are not. The median rLTL was significantly higher for participants who were currently active (t = -2.228, p = 0.013). However, the differences were rather small. This was not surprising, as the categorisation “currently active” considers neither the length of the activity period nor the type of activity practised.

Regarding the types of sport currently practised, Fig 2 provides some interesting insights as the one-way ANOVA revealed significant differences (F = 6.08, p < 0.001). The graphs for inactivity, resistance training, endurance and other types of sport showed fairly similar patterns, while individuals who engaged in intensive activity showed slightly higher values of rLTL. The *post hoc* Tukey’s test revealed significant differences for respondents engaged in intensive activity compared to inactive respondents (p = 0.011), and respondents who engaged in resistance training (p = 0.021): intensive activity is related to significant longer telomeres compared to these types of sport.

The period since when the current activity was practised was considered in Fig 3. The comparison of the four groups revealed a slightly, but significant, higher rLTL with increasing duration of physical activity (F = 4.39, p = 0.0048). The *post hoc* Tukey’s test revealed significant difference between the most active group (more than 41 years) and the current inactive group (p = 0.017) as well as the group physically active since 1 to 9 years (p = 0.035).

In contrast, physical activity practiced only between the age of 20 and 30 years does not associate with rLTL in later life. Fig 4 displays even slightly shorter rLTL for those individuals who have exercised in young age between 20 and 30 compared to those individuals who have never practiced sports. However, this difference is not significant (t = 0.358, p = 0.721).

The multivariate analysis confirmed the results of these bivariate distributions. The multiple regressions enabled us to estimate the impact of physical activity aspects on relative telomere length in dependence of potentially influential covariates. Descriptives of covariates according to sports types currently practiced are shown in Table A in S1 File. Since our different variables regarding physical activity are dependent upon each other, we decided to estimate separate models for each variable (Table 2). Model 1 of Table 2 displays the results of the basic regression model including only control variables but no information on physical activity. We found a significant effect of gender on relative telomere length. Male participants showed significantly longer telomeres compared to female participants. This effect remained constant across regression models.

**Table 2:** Regression Models for Relative Telomere Length (rLTL).

Variables	Model 1^a^	Model 2^b^	Model 3^c^	Model 4^d^	Model 5^e^
Age	0.00	0.01	0.01	0.04	0.01
	(0.01)	(0.01)	(0.01)	(0.03)	(0.02)
Male	0.55***	0.58***	0.53***	0.45*	0.41***
	(0.08)	(0.08)	(0.08)	(0.22)	(0.12)
Married	-0.10	-0.11	-0.10	-0.08	-0.07
	(0.08)	(0.08)	(0.08)	(0.23)	(0.13)
Father’s age at birth	-0.00	-0.00	-0.00	-0.01	-0.00
	(0.01)	(0.01)	(0.01)	(0.01)	(0.01)
Years of education	0.03	0.03	0.04	0.01	0.05
	(0.03)	(0.03)	(0.03)	(0.08)	(0.05)
Income	0.07	0.03	0.01	0.36	0.03
	(0.08)	(0.08)	(0.08)	(0.18)	(0.12)
Body mass index	-0.00	0.00	0.00	-0.02	0.01
	(0.01)	(0.01)	(0.01)	(0.02)	(0.01)
Alcohol intake above cut-off value by DHS	-0.07	-0.06	-0.08	0.02	-0.05
	(0.07)	(0.07)	(0.07)	(0.18)	(0.11)
Smoker	0.03	0.06	0.10	0.50	0.27
	(0.15)	(0.15)	(0.15)	(0.32)	(0.20)
Physically active		0.26***			
		(0.08)			
Type of physical activity (Reference: no sports)					
intensive activity			0.79***		
			(0.20)		
resistance training			0.18		
			(0.09)		
Endurance			0.26*		
			(0.10)		
other types of sports			0.24* (0.11)		
Physically active only in the age of 20–30 (vs. never active)				-0.16 (0.21)	
Duration since activity is practiced (Reference: no sports)					
Physically active for 1 to 9 years					0.00 (0.17)
Physically active for 10 to 41 years					0.39* (0.15)
Physically active for 42 to 72 years					0.47*** (0.14)
Constant	-0.98	-1.15	-1.09	-5.17	-1.36
	(1.08)	(1.08)	(1.08)	(3.01)	(1.51)
Observations	681	679	676	112	310
R^2^	0.08	0.10	0.11	0.13	0.10

Note: Standard errors in parentheses. Significance levels

*** p<0.001

** p<0.01

* p<0.5.

^a^ Model 1 refers to a regression for Relative Telomere Length (rLTL) including the basic covariates set: age, gender, married, father’s age at birth, years of education, income, body mass index, Alcohol intake above cut-off value by DHS, smoking status.

^b^ Model 2 refers to a regression for Relative Telomere Length (rLTL) including the basic covariates set + current physical activity status.

^c^ Model 3 refers to a regression for Relative Telomere Length (rLTL) including the basic covariates set +type of physical activity currently practiced.

^d^ Model 4 refers to a regression for Relative Telomere Length (rLTL) including the basic covariates set + physical activity status regarding the age 20–30.

^e^ Model 5 refers to a regression for Relative Telomere Length (rLTL) including the basic covariates set + the duration since the current physical activity is practiced.

We did not find any association between participants’ age, marital status, father’s age at birth, years of education, income, body mass index, alcohol intake above cut-off value defined by DHS, or current smoking status and the participant’s relative telomere length.

In model 2, we added the status of current physical activity, which turned out to be positively correlated with relative telomere length (β = 0.26, p < 0.001). In contrast, physical activity performed only during the age of 20 to 30 (model 4) compared to a totally sport-free lifestyle did not affect relative telomere length in later life (> 60 years). However, model 4 centered on a specific subpopulation of the BASE-II participants, namely those who were not currently exercising. Due to this focus, the sample size was reduced in this regression model and comparisons with the other models should be made with caution.

As displayed in model 5, the years of exercise influence relative telomere length. Remarkably, practicing sports for less than 10 years does not have a significant effect on relative telomere length (β = 0.00, p = 0.981). The variable reflecting a minimum of 42 years of physical activity showed the strongest association with relative telomere length (β = 0.47, p = 0.001). Regarding the types of sport, model 3 displays a strong positive association of intensive activity (β = 0.79, p < 0.001) and the significant but comparatively weaker impact of endurance sport with relative telomere length (β = 0.26, p = 0.011). In contrast, types of sport that can be defined as resistance training do not impact relative telomere length.

After adjusting for morbidity, as measured by an index largely based on the domains of the Charlson Index, the results remain stable across all models. These models are provided in Table B in S1 File. Overall, our regression models explain about 10% of variation in the relative telomere length.

## Discussion

In the current study, we have assessed the rLTL of 814 BASE-II participants aged 61 and older in relation to lifestyle factors that, in the literature, are associated with telomere length. We particularly focused on physical activity and sport, investigating the relationship between current sporting activity, sport in different stages of life, and rLTL. As described earlier [18], BASE-II men showed significantly longer telomeres than women, even after adjustment for known co-variates, a finding most likely explained by the method used for rLTL estimation ([18, 19]). We have previously shown that BASE-II participants of the older group (from which the subjects of the current analysis were drawn) had significantly shorter rLTL when compared to the younger group of BASE-II participants [18]. The fact that the participants’ age was not a significant determinant of rLTL in the current study is likely due to the narrow age range of the subjects studied.

Similar to several other studies, physical activity and sport were among the lifestyle factors positively associated with rLTL in the current study. Looking at different types of sport clearly revealed a closer association between intensive activity sports and rLTL, when compared to the degree of association between endurance sports and rLTL. Earlier studies on endurance exercise and telomere length have yielded conflicting results (reviewed in [20]). Examination of 17 habitually exercising and 15 sedentary older study participants revealed longer LTL (estimated by Southern blot) in the exercising group [21]. Denham and colleagues found significantly longer telomeres (rLTL) in 67 male ultra-marathon runners when compared to rLTL of 56 male controls [22]. Similarly, two other studies compared relative telomere length of endurance athletes with sedentary subjects [23] or subjects exercising at a medium level [24] and found longer telomeres in the athletes groups. These findings are contrasted by results from Rae et al., who found no difference in mean LTL (estimated by Southern blot) between 18 experienced endurance runners (42 ± 7 years of age) and 19 sedentary controls (39 ± 10 years of age) [25]. Mathur and colleagues also found no difference between 17 marathon runners and 15 age- and sex-matched healthy, sedentary control subjects [26]. A drawback of most of these and other similar studies is the small sample size, because this increases the risk that unconsidered confounders might have impacted on the results. While our study did not explicitly include marathon runners, we were able to distinguish endurance exercising participants from subjects pursuing sports at other activity levels using this large BASE-II sample of community-dwelling older participants. In addition, we were able to consider many of the known covariates such as lifestyle factors, the father’s age at birth and socio-economic factors.

One limitation of our study is that the data on physical activity and sports were self-reported and might therefore be biased. This bias might be of particular relevance where the interval between physical activity and self-report is especially long. Another drawback is the cross-sectional nature of the current analysis which does not allow any causal inferences.

It seems, however, plausible that the association between physical activity and telomere length, as frequently described in the literature and also found in the BASE-II participants studied here, reflects the individual histories of physical activity and their impact on telomere length, and this is what is also suggested by our results, at least to some degree. In a study performed by Savela and colleagues physical activity in midlife was associated with telomere length (southern blot) in old age [27]. However, the comparability between this investigation and our study is limited because the authors used the information on physical activity assessed in midlife of the study population and the telomere length data measured in old age of the participants with no further information on continuity of physical activity between the two time points.

The variables “exercise duration” with the outcomes “currently not active”, “physically active for 1 to 9 years”, “physically active for 10 to 41 years” and “physically active more than 41 years” were significantly associated with rLTL in our study. More interestingly, our data suggest that at least 10 years of regular sports activity is needed in order to impact rLTL.

Physical activity performed only during young adulthood (age between 20–30 years), however, was not predictive for rLTL in old age. This was surprising in the context of the idea that the number of cell divisions and, as a prerequisite, the number of DNA replications (associated with telomere shortening as a consequence of each replication), is influenced by lifestyle factors and that less cellular regenerative activity, e.g. because of less oxidative stress, is needed in individuals with health-conscious behavior, resulting in less telomere shortening. This would implicate a ‘cellular memory’ of lifestyle during different stages of life in the form of longer/shorter telomeres. Our data, however, do not support this view, since sport only in early adulthood was not predictive for rLTL in advanced age. This observation goes along with findings of a publication using data from the *Copenhagen City Heart Study*. In that study, physical activity and other lifestyle factors were significantly associated with telomere length, both measured twice with a 10 years interval. The change of telomere length during the 10 years, however, was not associated with baseline and inter-observational physical activity, and also not with other lifestyle factors investigated [7]. The authors speculate that underrepresentation of participants with short telomeres may have biased the analysis, explaining at least in part the strong associations between lifestyle factors and telomere length observed cross-sectionally.

In the context of our results we agree with this view and hypothesize that another part of the explanation is that physical activity indeed impacts on telomere length; however, with the limitation that only the 10 years prior the point of investigation are of relevance with respect to telomere length, with probably similar time frames for other lifestyle factors. It is not yet clear which mechanism(s) drive the impact of physical activity on telomere length. It might be less regenerative activity or increased enzymatic activity (or a combination of both) that decelerate telomere shortening or even result in telomere extension as an effect of physical activity. Indeed, there is some evidence for the latter in the literature [28].

The fact that the impact of physical activity on telomere length is limited and fades due to periods of inactivity might also have an evolutionary aspect. All studies on physical activity and telomere length are performed on populations who differ dramatically in their lifestyle from prehistoric man [28]. During evolution, humans adapted to the requirements of hunting and gathering, including a higher level of regular physical activity and this is what humans are still adapted to. If telomere length can be positively influenced by physical activity, it seems reasonable that this influence is limited in order not to turn it to a negative effect.

In conclusion we provide further cross-sectional evidence for a beneficial role of physical activity and sports with respect to telomere length. Our retrospective view on the association between physical activity pursued in different stages of life and telomere length indicated that this association becomes visible following at least 10 years of regular physical activity prior to the examination. Our results, however, also suggest that continuous physical activity is necessary to achieve a sustained effect on telomere length throughout life.

## Materials and Methods

BASE-II is a study on healthy aging covering residents of the greater metropolitan area of Berlin, Germany. The sample consists of a cohort of about 1,600 older individuals (aged 60–75 in 2009) and a cohort of 600 younger individuals (aged 20–35 in 2009) (for a detailed description of the study see [29]). For this analysis, we included all individuals who were over 60 years of age in 2012 and who participated both in the medical examination and the socio-economic module (for a description of this module which is a related study of the Socio-Economic Panel Study (SOEP) see [30]). This results in a final sample size of 814 participants. All participants gave written informed consent and the Ethics Committee of the Charité-Universitätsmedizin Berlin approved the study (approval number EA2/029/09).

### Relative leukocyte telomere length (rLTL)

The measurement of rLTL in BASE-II was described previously in detail [18]. Briefly, genomic DNA was extracted from EDTA blood using the LGC ‘Plus XL manual kit’ (LGC, Germany, Berlin). rLTL was measured using a modified quantitative PCR protocol originally described by Cawthon [31]. All samples were measured in triplicate and their mean was used for further analysis when the ct values of both PCRs (telomere PCR and single copy gene [36B4] PCR) showed a variation coefficient < 2%. The rLTL was subsequently calculated according to Pfaffl et al. [32]. Pooled DNA from 10 randomly selected subjects was used as the reference (rLTL = 1).

### Physical activity

Information on physical activity was taken from the 2012 socio-economic survey of BASE-II, where participants were asked whether they currently practise a sport, what type of sport they practise, and when they started practising this type of sport. Additionally, it was surveyed whether respondents practised another type of sport in the past or had ever engaged in physical activity. The year of the beginning and the end of the physical activity was also documented. This information enabled us to generate a variety of indicators of physical activity. For our analysis we used the following indicators: *(i)* current status of physical activity (no/yes); *(ii)* type of sport currently practised (0 = no sport; 1 = intensive activity e.g. badminton, basketball, skiing; 2 = resistance training, e.g. bodybuilding, gymnastics; 3 = endurance, e.g. cycling, jogging, inline skating; 4 = other type of sport); *(iii)* whether the respondent only participated in sport in young adulthood (between the age of 20 to 30; 0 = no sport at all, 1 = sport in young adulthood); and *(iv)* for how long has the participant been exercising (0 = if not exercising currently, 1 = 1 to 9 years, 2 = 10 to 41 years, 3 = more than 41 years).

### Control variables

In addition to the indicators of physical activity, we controlled for age in years, gender, marital status, father’s age at birth, years of education and the logarithm of the equalized household income. For the purpose of calculating an equalized household income, each person of a household received a weight. While children received the weight 0.3, the first adult received the weight 1 and all other adults in the household received the weight 0.5. The equalized household income was then calculated by dividing the net household income by the weighted household size.

In addition, we included health-related indicators in the analysis, namely body mass index, current smoking status (no/yes), and alcohol intake above cut-off values defined by the German Centre for Addiction Issues (DHS). The DHS defines a cut-off value of 24 g per day for men and 12 g per day for women as non-hazardous alcohol intake [33]. Accordingly, we used a variable that is 1 for women who drank at least 12.5 g alcohol per day and for men who drank at least 25 g alcohol per day.

In order to allow controlling for morbidity we computed an index based on participant-reported and physician-observed medical diagnosis (for details see [34]). This morbidity index largely covers the categories and weighted classification scheme used by the *Charlson Index* [35].

### Statistical analysis

First, we compared column scatter graphs (GraphPad Prism 6) displaying the distribution of telomere length by different aspects regarding physical activity. We used either t-tests or ANOVA with post-hoc tests (Tukey’s test with the Tukey–Kramer adjustment for unequal sample sizes) to reveal potential differences between groups.

Second, we estimated five different multiple linear regression models, with robust standard errors to account for possible heteroscedasticity, in order to assess the relationship between physical activity and relative telomere length, while adjusting for a variety of covariates. We used the STATA statistical software package, version 13, for analysis.

The first model contains only the control variables. In the other models, we included different information on physical activity, namely, a binary variable indicating whether respondents currently exercise (model 2); the type of sport currently performed (model 3); a binary variable indicating whether respondents exercised only between 20 and 30 years of age, or did not exercise at all (model 4). In addition, we included information regarding exercise duration in model 5. The categorisation enabled us to see whether physical activity for a period of less than 10 years already associates with relative telomere length. Since the measurement of the relative telomere length was conducted on samples collected during a longer period of time (from 2009 to 2014), we were faced with the problem that telomere length information was in some cases newer than the information on physical activity. We run robustness checks to disentangle possible effects on our regression estimates. We could only observe differences for the duration of physical activity (model 5) in the effects. Therefore, we decided to run the regression for model 5 on the reduced sample, ignoring all cases where the telomere length was measured prior to 2012.

## Supporting Information

S1 File Table A. Descriptives of Covariates by Sports Types currently practiced. Table B. Regression Models for Relative Telomere Length (rLTL) controlling for Morbidity. (DOCX)
