# Supplementary material for: Sports and Exercise at Different Ages and Leukocyte Telomere Length in Later Life – Data from the Berlin Aging Study II (BASE-II)
Source: PLoS One. 2015 Dec 2;10(12):e0142131. doi: 10.1371/journal.pone.0142131 (PMC4668005; doi:10.1371/journal.pone.0142131)
Supplement: S1 File — (DOCX) [file pone.0142131.s001.docx]

**S1 File.**

**Table A. Descriptives of Covariates by Sports Types currently practiced**

|  | Total Observations | Male | Smoker | Heavy alcohol intake | Years of Education (mean) | BMI  (mean) |
| --- | --- | --- | --- | --- | --- | --- |
| Not active | 223 | 128 | 22 | 60 | 13.9 | 27.8 |
| Currently active | 453 | 200 | 28 | 127 | 13.9 | 26.4 |
| Intensive activity | 32 | 23 | 0 | 10 | 14.1 | 25.9 |
| Resistance training | 177 | 58 | 15 | 48 | 14.2 | 26.3 |
| Endurance | 138 | 73 | 10 | 38 | 14.0 | 26.2 |
| Other types of sports | 106 | 46 | 3 | 31 | 13.4 | 27.0 |
| Total | 676 | 328 | 50 | 187 | 13.9 | 26.9 |

**Table B. Regression Models for Relative Telomere Length (rLTL) controlling for Morbidity**

| Variables | Model 1^a^ | Model 2^b^ | Model 3^c^ | Model 4^d^ | Model 5^e^ |
| --- | --- | --- | --- | --- | --- |
| Age | 0.01 | 0.01 | 0.01 | 0.05 | 0.01 |
|  | (0.01) | (0.01) | (0.01) | (0.03) | (0.02) |
| Male | 0.55*** | 0.58*** | 0.53*** | 0.45* | 0.42*** |
|  | (0.08) | (0.08) | (0.08) | (0.22) | (0.12) |
| Married | -0.10 | -0.11 | -0.11 | -0.05 | -0.10 |
|  | (0.08) | (0.08) | (0.08) | (0.24) | (0.13) |
| Father’s age at birth | -0.00 | -0.00 | -0.00 | -0.01 | -0.00 |
|  | (0.01) | (0.01) | (0.01) | (0.01) | (0.01) |
| Years of education | 0.03 | 0.03 | 0.03 | 0.04 | 0.04 |
|  | (0.03) | (0.03) | (0.03) | (0.07) | (0.05) |
| Income | 0.06 | 0.02 | 0.01 | 0.39* | 0.01 |
|  | (0.08) | (0.08) | (0.08) | (0.18) | (0.12) |
| Body mass index | -0.00 | 0.00 | 0.01 | -0.02 | 0.01 |
|  | (0.01) | (0.01) | (0.01) | (0.02) | (0.01) |
| Alcohol intake above cut-off value by DHS | -0.07 | -0.07 | -0.08 | 0.08 | -0.04 |
|  | (0.07) | (0.07) | (0.07) | (0.20) | (0.11) |
| Smoker | 0.04 | 0.06 | 0.10 | 0.55 | 0.27 |
|  | (0.15) | (0.15) | (0.15) | (0.33) | (0.19) |
| Morbidity: low score | -0.10 | -0.09 | -0.08 | -0.39 | 0.02 |
| (Reference: score=0) | (0.09) | (0.09) | (0.09) | (0.28) | (0.15) |
| Morbidity: high score | 0.11 | 0.12 | 0.14 | 0.19 | 0.21 |
|  | (0.09) | (0.09) | (0.09) | (0.23) | (0.13) |
| Physically active |  | 0.27*** |  |  |  |
|  |  | (0.08) |  |  |  |
| Type of physical activity (Reference: no sports) |  |  |  |  |  |
| intensive activity |  |  | 0.80*** |  |  |
|  |  |  | (0.19) |  |  |
| resistance training |  |  | 0.18* |  |  |
|  |  |  | (0.09) |  |  |
| Endurance |  |  | 0.26* |  |  |
|  |  |  | (0.10) |  |  |
| other types of sports |  |  | 0.24*  (0.12) |  |  |
| Physically active only in the age of 20-30 (vs. never active) |  |  |  | -0.14  (0.21) |  |
| Duration since activity is practiced (Reference: no sports) |  |  |  |  |  |
| Physically active for 1 to 9 years |  |  |  |  | 0.01  (0.18) |
| Physically active for 10 to 41 years |  |  |  |  | 0.39**  (0.15) |
| Physically active for 42 to 72 years |  |  |  |  | 0.48***  (0.14) |
| Constant | -0.99 | -1.17 | -1.10 | -5.83 | -1.28 |
|  | (1.08) | (1.08) | (1.08) | (2.98) | (1.52) |
| Observations | 681 | 679 | 676 | 112 | 310 |
| R² | 0.08 | 0.10 | 0.12 | 0.16 | 0.10 |

Note: Standard errors in parentheses. Significance levels: *** p<0.001, ** p<0.01, * p<0.5

^a^ Model 1 refers to a regression for Relative Telomere Length (rLTL) including the basic covariates set: age, gender, married, father’s age at birth, years of education, income, body mass index, Alcohol intake above cut-off value by DHS smoking status.

^b^ Model 2 refers to a regression for Relative Telomere Length (rLTL) including the basic covariates set + current physical activity status

^c^ Model 3 refers to a regression for Relative Telomere Length (rLTL) including the basic covariates set +type of physical activity currently practiced.

^d^ Model 4 refers to a regression for Relative Telomere Length (rLTL) including the basic covariates set + physical activity status regarding the age 20-30.

^e^ Model 5 refers to a regression for Relative Telomere Length (rLTL) including the basic covariates set + the duration since the current physical activity is practiced.
